# The Prevalence and Diversity of Marine Toxin–Antitoxin Systems

**DOI:** 10.3390/md23110436

**Published:** 2025-11-13

**Authors:** Cong Liu, Yunxue Guo, Jiayu Gu, Zhen Wei, Pengxiang Chen, Xiaoxue Wang

**Affiliations:** 1 State Key Laboratory of Tropical Oceanography, South China Sea Institute of Oceanology, Chinese Academy of Sciences, Guangzhou 510301, China; liucong23@mails.ucas.ac.cn (C.L.); gujiayu20@mails.ucas.ac.cn (J.G.); 18238650981@163.com (Z.W.); chenpengxiang24@mails.ucas.ac.cn (P.C.); 2 University of Chinese Academy of Sciences, Beijing 100049, China

**Keywords:** toxin–antitoxin system, marine bacteria, bioinformatics, evolutionary divergence, environmental adaptation

## Abstract

Toxin-antitoxin (TA) systems, ubiquitous in bacterial and archaeal genomes, play pivotal roles in responding to environmental stresses, forming biofilms, defending against phages, and influencing pathogen virulence. The marine environment harbors Earth’s most diverse and abundant microbial communities, where microorganisms have evolved unique genetic adaptations and specialized metabolic processes to thrive amid distinct environmental challenges. Research on the presence and function of TA systems in marine bacteria lags significantly behind that in model bacteria and pathogens. Here, we explored the diversity of the TA system in marine bacteria, including species from the Global Ocean Microbiome Catalogue (GOMC) and the Mariana Trench Environment and Ecology Research (MEER) databases. Our findings revealed that types I to VII (featuring protein toxins) of eight types of TA systems are prevalent in these microorganisms, with unidentified TA combinations diverging from previously characterized systems. Interestingly, some toxins or antitoxins lack canonical counterparts, indicating evolutionary divergence. Additionally, previously uncharacterized potential TA systems have been identified in extremophilic bacteria from the deep-sea Mariana Trench. These results highlight the adaptive importance of marine TA systems, which are likely operating through unconventional mechanisms.

## 1. Introduction

**Figure 1:**
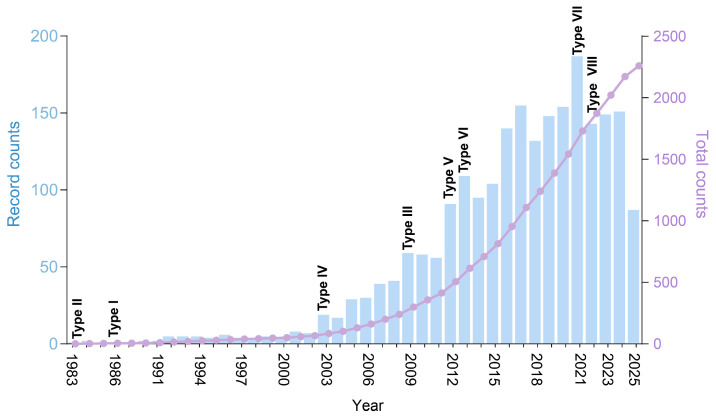
Analysis of publications on TA systems. Annual publication counts (bars) and cumulative publications (lines) from 1983 to 22 September 2025. Key years marking the identification of major TA system types are labeled.

Toxin-antitoxin (TA) systems represent prevalent genetic elements in bacteria and archaea and consist of two core components: a stable toxin that interferes with essential cellular processes—such as DNA replication, translation, or cell wall synthesis—and an inherently unstable antitoxin that neutralizes the toxin’s effects [1,2,3,4,5]. The significance of TA systems has gained increasing attention over the past four decades, as evidenced by both a consistent annual increase in related publications (Figure 1) and their identification in previously unexplored environmental niches. Currently, these systems are classified into eight distinct types (I–VIII) on the basis of the molecular nature of the TA systems and their modes of neutralization [2,5]. The mechanisms through which antitoxins counteract toxins align with this classification, reflecting remarkable mechanical diversity between two or three neighboring genes. In extensively studied type II TA systems, the protein antitoxins sterically block the toxin’s active site or disrupt its interaction with substrates via direct protein-protein interactions [6]. Type I and type III TA systems utilize antisense RNA or RNA antitoxins, respectively, to inhibit toxin synthesis or function through sequence-specific base pairing or structural complementarity [7,8,9]. Type IV antitoxins safeguard cellular targets rather than directly interacting with toxins, whereas type V antitoxins function as endoribonucleases that degrade toxin-encoding mRNAs [10]. Type VI TA systems utilize proteolytic antitoxins to degrade toxin proteins [11], whereas type VII antitoxins neutralize toxins via direct posttranslational modifications [12,13,14,15]. Notably, in type VIII TA systems, both toxins and antitoxins are RNA molecules. Two examples have been identified: (1) the antitoxin RyeA and toxin SdsR, which bind to form a duplex that is degraded by RNase III [16], and (2) the antitoxin CreA, which partners with the CRISPR-associated Cascade complex to transcriptionally repress the toxin gene *creT* [17]. Understanding these mechanisms not only advances our comprehension of bacterial resilience but also provides a foundation for developing strategies aimed at disrupting TA systems, thereby eliminating the killing or growth-inhibiting activity of toxins, as reflected by their associated physiological impacts.

**Figure 2:**
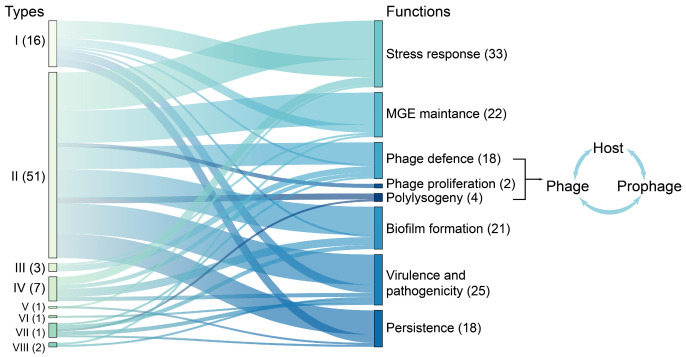
Functionally validated TA system types. Bar length denotes publication volume; edge width scales with co-occurrence frequency. Functions, including phage defense, phage proliferation, and polylysogeny, are involved in host–phage–prophage interactions. The numbers represent the abundance of functionally validated TA systems used for generating the figure.

TA systems play crucial roles in governing bacterial responses to environmental stressors [6], such as nutrient deprivation, antibiotic exposure, and immune attacks. Apart from their stress-responsive functions, TA systems are implicated in diverse processes (summarized in [2,3]), including the maintenance of mobile genetic elements (MGEs), biofilm formation, the modulation of virulence and pathogenicity, the intricate interplay among host–phage–prophage interactions, and the controversial phenomenon of bacterial persistence, which is correlated with antibiotic tolerance and chronic infections (Figure 2). Despite the ongoing debate, most recently, the SehA/B and RelE/B TA systems have been identified as facilitators of the persistent phenotype in *Salmonella* [18]. Moreover, the type I toxin TisB triggers the formation of persister cells by fully dissipating the proton gradient, thereby significantly impeding ATP production subsequent to antibiotic-induced DNA damage [19]. Understanding the molecular intricacies of TA systems not only enhances our understanding of bacterial survival strategies but also reveals avenues for novel therapeutic interventions targeting persistent infections, potentially overcoming antibiotic resistance. As research has advanced, TA systems have emerged as promising targets for combating persistent infections, highlighting their importance in both fundamental microbiology and applied medical science.

The majority of identified TA systems have been extensively characterized in model strains such as *Escherichia coli* K-12 and various pathogens such as *Mycobacterium tuberculosis* and *Pseudomonas aeruginosa,* where there is a paucity of reports concerning their presence in environmental microbiology contexts. Marine bacteria thrive in some of Earth’s most extreme environments, ranging from hydrothermal vents and polar ice to deep-sea trenches and hypersaline basins. These challenging habitats, which are characterized by extreme conditions of temperature, pressure, salinity, and nutrient scarcity, have driven the evolution of unique physiological and metabolic traits within marine microbial communities. In this study, we systematically investigated the genomic landscape of TA systems across diverse marine bacteria, leveraging metagenome-assembled genomes (MAGs) from the Global Ocean Microbiome Catalogue (GOMC) [20] and Mariana Trench Environment and Ecology Research (MEER) microbial databases [21]. We reveal a remarkable diversity of TA systems, including novel noncanonical pairings and abundant orphan components, which are particularly enriched in deep-sea extremophiles. These findings demonstrate that marine TA systems exhibit distinct evolutionary patterns and environmental specificity, likely underpinning adaptations to extreme marine habitats, such as high hydrostatic pressure, low temperature, and nutrient scarcity.

## 2. Results and Discussion

### 2.1. TA Systems Are Prevalent in Marine Microorganisms with Divergent Abundance

Marine microbial communities are fundamental drivers of global biogeochemical cycles and harbor vast potential for new genetic resources [22]. Genomic initiatives such as the GOMC, which assembles 24,195 species-level genomes spanning 138 phyla, represent the comprehensive resources for functional gene mining in marine microbes, including TA systems [23]. Marine ecosystems span a continuum of habitats, from shallow photosynthetic zones to deep-sea hydrothermal vents (350–400 °C, pH 2–3, metal/H_2_S-rich) [24,25,26] and hadal trenches (6000–11,000 m, >100 MPa, darkness, oligotrophic) [27,28], featuring the gradients of high salinity, hydrostatic pressure (HHP) gradients, low temperature (LT) and thermal stratification [20]. These factors drive microbial evolutionary adaptations such as streamlining genome size and featuring unique metabolic capabilities [21]. Notably, the MEER characterized 7564 species (89.4% novel) and revealed specialized antioxidative gene repertoires and ubiquitous nutrient cycling (C, N, S, H) in deep-sea prokaryotes [21].

**Figure 3:**
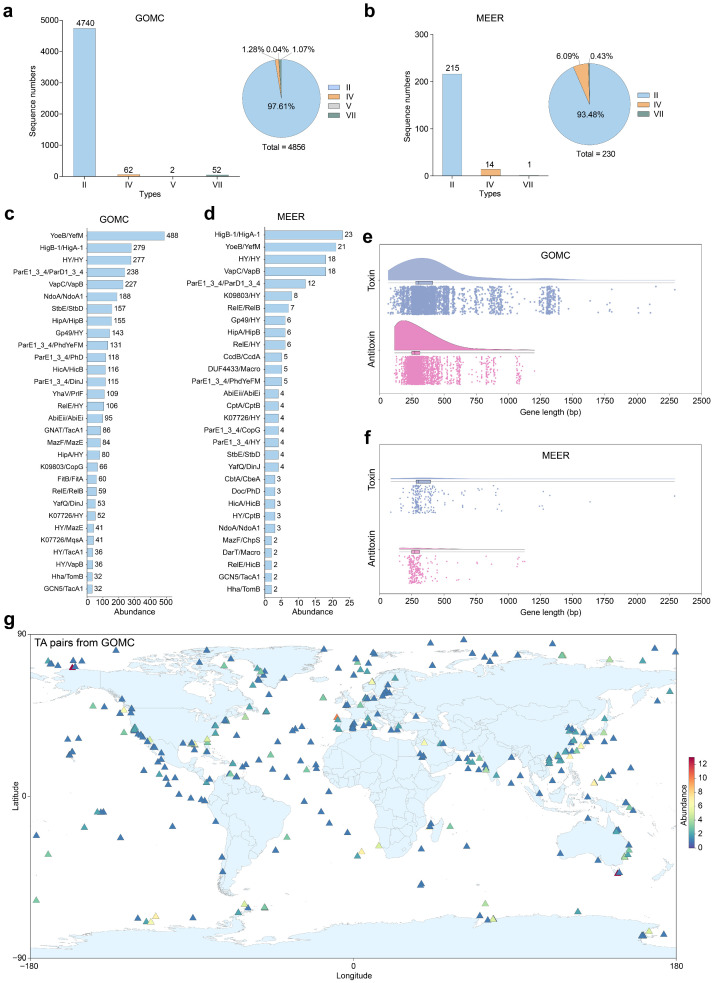
Genomic features and biogeographic distribution of marine toxin–antitoxin (TA) systems. Stacked bar charts and adjacent pie charts showing the TA type-specific proportional compositions of Global Ocean Microbiome Catalogue (GOMC) (*n* = 24,195 genomes) (**a**) and Mariana Trench Environment and Ecology Research (MEER) microbial databases (*n* = 7564 MAGs) (**b**). Relative abundance of the top 30 TA systems in the GOMC (**c**) and MEER (**d**) databases, where hypothetical proteins are designated “HY”. Box-and-whisker plots display gene length distributions for toxin/antitoxin components in the GOMC (**e**) and MEER (**f**) databases, with centerlines indicating medians and boxes spanning the 25th–75th percentiles. (**g**) Global biogeographic map (Equirectangular projection, EPSG: 4326) visualizing TA-harboring prokaryotes via coordinates sourced from the GOMC and Ocean Microbiome Database (OMD) [23], with relative abundance gradients encoded on a chromatic scale. The identified TA systems are listed in Table S1.

Although environmental adaptation-related TA systems have been identified in several marine bacterial species [29,30,31,32,33], they remain systematically underexplored. To comprehensively delineate the landscape and diversity of TA systems across marine bacterial and archaeal habitats, from surface seawater to hadal zones, we conducted TA locus predictions in MAGs sourced from the GOMC and MEER databases via TADB 3.0 [34]. Our analysis revealed 4856 TA systems in 2179 MAGs (2152 bacterial, 27 archaeal) of the GOMC (Figure 3a), demonstrating their pervasive distribution in marine microbiomes. The bacterial MAGs hosted 4807 systems primarily featuring biotechnologically pertinent type II TA systems (4693/4807; 97.63%), with lower occurrences of types IV (60), VII (52) and V (2), whereas the archaeal MAGs represented only 49 systems. Notably, complete TA systems (230) were concentrated in only 116 bacterial MAGs of MEER, showing a notable decrease in abundance within deep-sea prokaryotic community (230/7564 genomes) as a whole compared with shallower counterparts (4856/24,159 genomes), indicating distinct evolutionary pressures in extreme hadal environments (Figure 3b). Among the TA systems predicted in both GOMC and MEER, the top five systems were identified the same as YoeB/YefM, HigB-1/HigA-1, ParE1_3_4/ParD1_3_4, VapC/VapB, and a pair involving two hypothetical proteins (Figure 3c,d). Notably, deep-sea bacteria exclusively maintained types II (215/230; 93.48%), IV (14), and VII (1) TA systems, a streamlined repertoire potentially optimized for high-stress adaptation. The majority of toxin/antitoxin genes displayed compact 200–500 bp architectures (Figure 3e,f), which are advantageous for genetic engineering applications. To assess the potential geographical clustering of TA systems across global oceans, we mapped all characterized TA systems from the GOMC database alongside the sampling sites. Spatial analysis revealed that global mapping confirmed the nearly ubiquitous distribution of TA systems throughout marine ecosystems with no significant regional aggregation patterns observed (Figure 3g). The results underscored their dual importance: as fundamental stress–response models by extreme habitats and as untapped biotechnological reservoirs with properties such as heat stability, cryotolerance, and pressure resilience that hold promise for industrial enzyme design, antimicrobial development, and synthetic biology.

**Figure 4:**
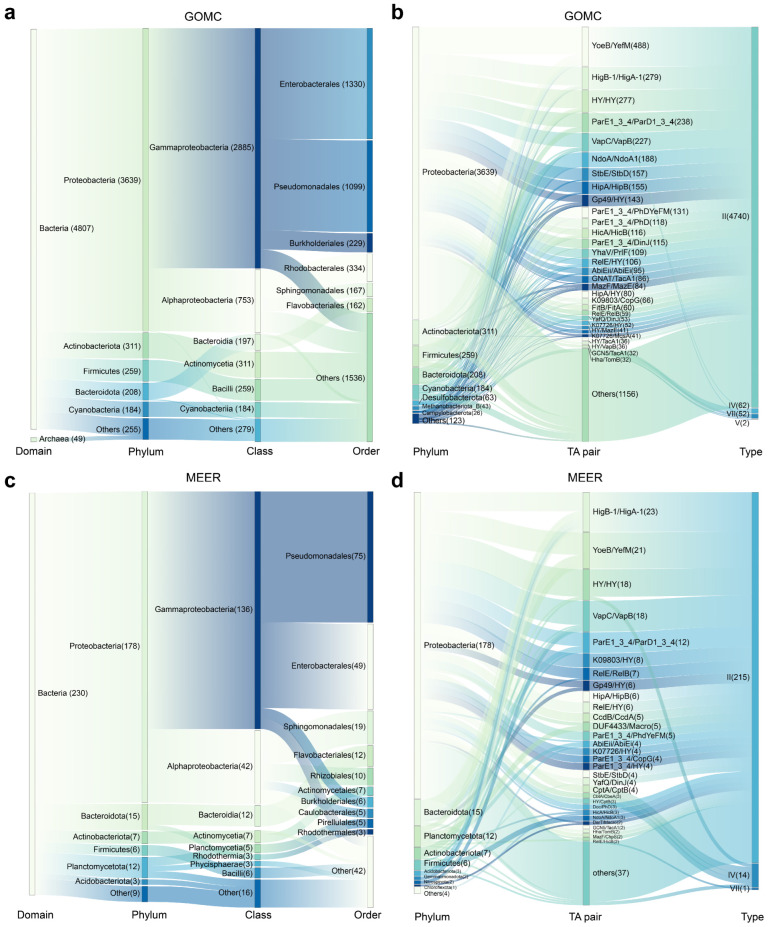
Host-taxonomic associations of marine TA systems. Sankey diagrams mapping the hierarchical taxonomy of TA-harboring prokaryotes (**a**) and the phylum-level host distributions of the top 30 TA systems (**b**) in the GOMC database. The flow volumes are scaled to relative abundance, and the connector widths encode pairwise association strength. Sankey diagrams mapping the hierarchical taxonomy of TA-harboring prokaryotes (**c**) and the phylum-level host distributions of the top 30 TA systems (**d**) in the MEER database. All the abundance metrics represent relative proportions calculated from the predicted results.

We next investigated the distribution patterns of TA systems across bacterial taxa. Within the GOMC database, *Proteobacteria* dominated the abundance of TA systems with 3639 systems, together with *Actinobacteriota* (311), *Firmicutes* (259), *Bacteroidota* (208) and *Cyanobacteria* (184), collectively accounting for 95.71% (4601/4807) of all bacterial TA systems (Figure 4a,b). The diverse TA systems were also identified in the species across bacterial and archaeal MAGs from the GOMC and MEER databases, and many were not previously reported to harbor TA systems (Table 1). In the MEER database, TA systems were consistently detected throughout the hadal depth range (6000–11,000 m), though their abundance did not correlate with sampling depth (Table 1). Notably, the GOMC database highlighted a pronounced prevalence of type II TA systems within specific prokaryotic lineages (Table 1). For example, the denitrifying bacterium *Marinobacter* sp. Arc7-DN-1 (GOMC-BGIocean__5042), which was isolated from Arctic Ocean sediment, harbored 18 type II TA systems. Similarly, the pathogens *Vibrio cholerae* (GOMC-BGIocean__2532) and *Pantoea agglomerans* (GOMC-BGIocean__2698) each carried 17 type II systems. In contrast, the MEER database revealed significant phylum-level divergences: *Proteobacteria* harbored 77.4% of the TA systems (178/230), whereas phyla such as *Bacteroidota* and *Planctomycetota* contained <20% of the systems despite their high prevalence (Figure 4c,d). This skewed distribution implies a specialized evolutionary retention of TA modules under extreme conditions. However, three exceptional strains harbor >10 TA systems were identified at species level—*Acinetobacter* sp. (strain MEER__7415) featured 16 type II systems; *Citrobacter freundii* (MEER__7435) possessed 7 type II systems and 4 type IV systems; and *Klebsiella pneumoniae* (MEER__3538) carried 10 type II systems and 1 type IV system. Interestingly, these high-TA carriers are both opportunistic human pathogens and have various environmental functions—*C. freundii*, for example, plays a key role in nitrate reduction, which is essential for deep-sea nitrogen cycling. This dual role positions them as exceptional targets for bioprospecting: their TA systems likely facilitate adaptation to diverse stressors, potentially leveraging regulatory pathways inherited from pathogenic lineages. Therefore, deep-sea TA networks, particularly the biotechnologically adaptable type II systems prevalent in marine microbiomes, represent unexplored reservoirs for (1) developing novel antimicrobial adjuncts that leverage conserved stress–response mechanisms, (2) designing pressure-tolerant genetic circuits for industrial biocatalysis, and (3) harnessing regulatory modules derived from pathogens for synthetic biology applications.

### 2.2. Novel and Diverse TA Combinations for Marine Microorganisms Differing from Characterized Systems

While the majority of identified TA systems (97.42%; 4955/5086) adhered to canonical type II architectures, primarily YoeB/YefM, HigB-1/HigA-1, ParE1-3-4/ParD1-3-4, and VapC/VapB, marine prokaryotes displayed significant non-canonical diversity. Our analysis also uncovered a prevalent class of hypothetical toxins/antitoxins (designated as HY), including 295 HY/HY (277 from GOMC, 18 from MEER) domain combinations. While 74.77% (403/539) of the hypothetical toxins remained unclassified, the antitoxins were more amenable. Successful re-annotation of antitoxins (715/929, 76.97%) revealed a predominance of the HTH family (344/929, 37.03%) and dnstrm_H1420 domain-containing proteins (131/929, 14.10%) (Table S1). Notably, toxins with well-defined domains (e.g., ParE1_3_4, RelE, GNAT, HipA, and VapC) often pair with antitoxins carrying uncharacterized domains such as DNA-binding proteins, transcriptional regulators, and helix-turn-helix proteins of unknown function. Newly identified combinations included the integration of PIN (PilT N-terminal) domains (RNA-binding) with DNA-binding motifs (Figure 3c, Table 2), indicating adaptations specific to certain niches. Moreover, we revealed unexpected modularity, where canonical toxins (e.g., ParE1_3_4) were linked with noncognate antitoxins (Phd, DinJ and CopG), as well as entirely novel pairings (e.g., FitB/Phd, StbE/YefM, RelE/StbD and MazF/PrlF) (Table 2). The only type VII (bacterial) TA system in the MEER database was HY/HY, with additional novel combinations (ParE1_3_4/CopG, ParE1_3_4/DinJ and YhaV/PrlF) highlighting adaptive innovation.

**Figure 5:**
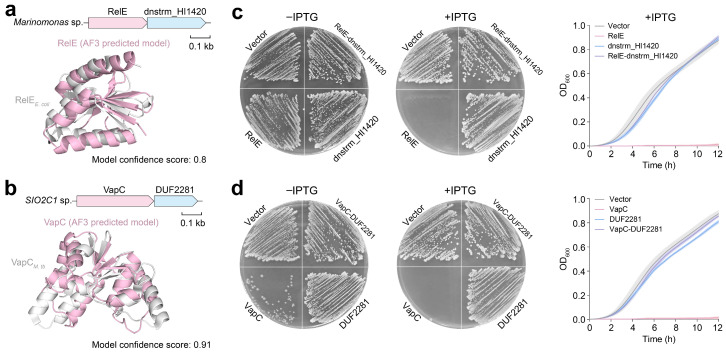
Experimental validation of novel TA systems. (**a**) Structure alignment between RelE homologs from *E. coli* (PDB code: 4FXI) and *Marinomonas* sp. (AlphaFold 3 model confidence scores: 0.8). (**b**) Structure alignment between VapC homologs from *M. tuberculosis* (PDB code: 3DBO) and *SIO2C1* sp. (AlphaFold 3 model confidence scores: 0.91). Toxicity assay of the RelE/dnstrm_HI1420 system from *Marinomonas* sp. (**c**) and the VapC/DUF2281 system from *SIO2C1* sp. (**d**). Gene expression was induced by 0.2 mM IPTG, demonstrating the lethal effect of the toxin and its neutralization by the cognate antitoxin. Representative images of the plates used for the toxicity assay are shown. For each growth experiment, three biological replicates are presented as individual curves, and each represents the means ± SDs of three technical replicates.

To experimentally validate the function of these novel pairings, we synthesized two candidate TA systems featuring hypothetical antitoxins that we named according to their domains—RelE/dnstrm_HI1420 and VapC/DUF2281. Among these candidates, the toxin components belong to the well-known RelE and VapC families (Figure 5a,b). We then compared these two toxins to their canonical counterparts. The RelE toxin functions as a ribonuclease that cleaves mRNA bound to the ribosome during translation stress [35]. It typically shares limited sequence similarity with its cognate antitoxin RelB, which neutralizes RelE through direct binding, often involving a flexible C-terminal region [36]. Similarly, VapC is a ribonuclease that targets specific tRNAs or mRNAs, while its conventional antitoxin VapB neutralizes VapC activity via protein–protein interactions, often forming an α-helix that occludes the VapC active site [37,38,39]. However, both dnstrm_HI1420 and DUF2281 show no significant sequence homology to RelB or VapB, indicating that they may represent evolutionarily distinct antitoxin lineages. Notably, both candidate systems display an inverted genomic arrangement compared with the typical RelBE and VapBC organizations, where the antitoxin gene usually precedes the toxin gene. Among our candidates, the toxin gene is located upstream of the antitoxin, a configuration previously linked to increased toxicity (Figure 5a,b).

We subsequently cloned the toxin genes, antitoxin genes, and full TA operons (Table S2) into an IPTG-inducible pTac vector and subsequently transformed them into *E. coli* MG1655 (Tables S3 and S4). Toxicity assays revealed that the overexpression of *relE* or *vapC* strongly inhibited bacterial growth, whereas the induction of *dnstrm_HI1420* or *DUF2281* alone had no toxic effect (Figure 5c,d). Co-expression of each antitoxin with its cognate toxin effectively neutralized the toxicity, confirming that both pairs constitute bona fide TA modules. Notably, VapC was toxic even in the absence of IPTG induction, indicating that its basal expression level is sufficient to cause toxicity and confirming its high potency. These findings suggest that marine microorganisms harbor diverse TA systems, including many uncharacterized variants, which may facilitate adaptation to extreme environments and represent valuable genetic resources for future exploration.

### 2.3. Orphan Toxins or Antitoxins Lacking Canonical Counterparts Are Abundant in Marine Microorganisms

**Figure 6:**
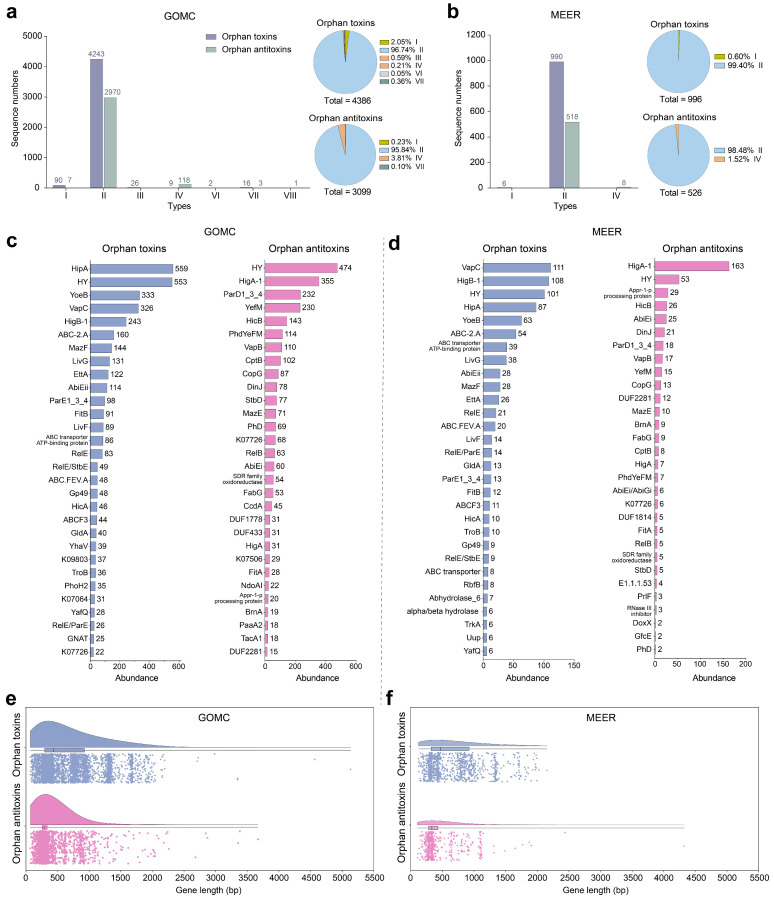
Genomic features and biogeographic distribution of orphan toxins and antitoxins. Stacked bar charts depict absolute counts of orphan toxins/antitoxins and adjacent pie charts showing type-specific proportional compositions in the GOMC (**a**) and MEER (**b**) databases. Relative abundance of the top 30 orphan toxins and antitoxins in the GOMC (**c**) and MEER (**d**) databases, where hypothetical proteins are designated “HY”. Box-and-whisker plots display gene length distributions for orphan toxins/antitoxins in the GOMC (**e**) and MEER (**f**) databases, with centerlines indicating medians and boxes spanning the 25th–75th percentiles. All orphan toxins/antitoxins are listed in Tables S5 and S6.

While canonical TA systems typically comprise cognate TA systems, our analysis revealed a widespread distribution of orphan toxins and antitoxins lacking canonical partners in marine microorganisms. We identified 4386 orphan toxins in 3038 MAGs (2980 bacterial, 58 archaeal) and 3099 orphan antitoxins in 2181 MAGs (2146 bacterial, 35 archaeal) in the GOMC without identifying their potential neighboring cognate partners (Figure 6a). Similarly, 4320 (98.50%) orphan toxins and 3059 (98.71%) orphan antitoxins occurred in the bacterial MAGs. However, different type-specific distributions between orphan toxins and antitoxins were revealed in the GOMC database. For the orphan toxins and antitoxins, the type II family dominated both categories, representing 96.74% (4243/4386) of the orphan toxins and 95.84% (2970/3099) of the antitoxins (Figure 6a). Type I toxins (90/4386, 2.05%) predominated over type II toxins, followed by type III (26), type VII (16), type IV (9) and type VI (2) toxins (Figure 6a). In contrast, orphan antitoxins showed strong type IV enrichment (118/3099, 3.81%), whereas type I enrichment was relatively rare (7/3099, 0.23%). Additionally, only one type VIII antitoxin was identified in the GOMC (Figure 6a). Interestingly, orphan toxins and antitoxins substantially outnumbered complete TA systems in deep-sea microbiomes. We identified 996 orphan toxins in 786 MAGs (777 bacterial, 9 archaeal) and 526 antitoxins in 455 MAGs (448 bacterial, 7 archaeal) in MEER (Figure 6b). Similarly, the type II family also dominated both the orphan toxins (990/996, 99.40%) and the antitoxins (518/526, 98.48%), with fewer occurrences of type IV orphan toxins (Figure 6b).

Notably, the top five orphan toxins, including HipA, VapC, YoeB, HigB-1 and HY, were derived from abundant TA systems in both GOMC and MEER databases. Among the orphan toxins, HipA, VapC, YoeB and HigB-1 represented a significant proportion (1461/4386, 33.31%) of the GOMC toxins, whereas they collectively represented 37.05% (369/996) of the MEER toxins (Figure 6c,d). Novel components, such as CopG, Gp49, FabG and DUF2281 family proteins, were also identified (Figure 6c). Prevalent orphan antitoxins, including HigA-1, ParD1_3_4, YefM, and HicB, were predominant (960/3099, 30.98%) in the GOMC. HigA-1 was the most abundant orphan antitoxins (163/526, 30.99%) in MEER (Figure 6c,d). Similar to the TA systems, hypothetical orphan toxins were largely unclassifiable (475/654, 72.63%). Conversely, 73.05% (385/527) of orphan antitoxins were re-annotated, revealing a clear predominance of the dnstrm_H1420 domain-containing (150/527, 28.46%) and HTH family proteins (79/527, 15.00%) (Tables S5 and S6). Most orphan antitoxin genes exhibited compact lengths of 200–500 bp, whereas orphan toxins were generally longer, spanning 250–1000 bp (Figure 6e,f). These results highlight many reservoirs and novel characteristics of non-canonical toxins/antitoxins in marine microbiomes. Functional validation of these orphan toxins/antitoxins remains essential for confirming their biological activities.

**Figure 7:**
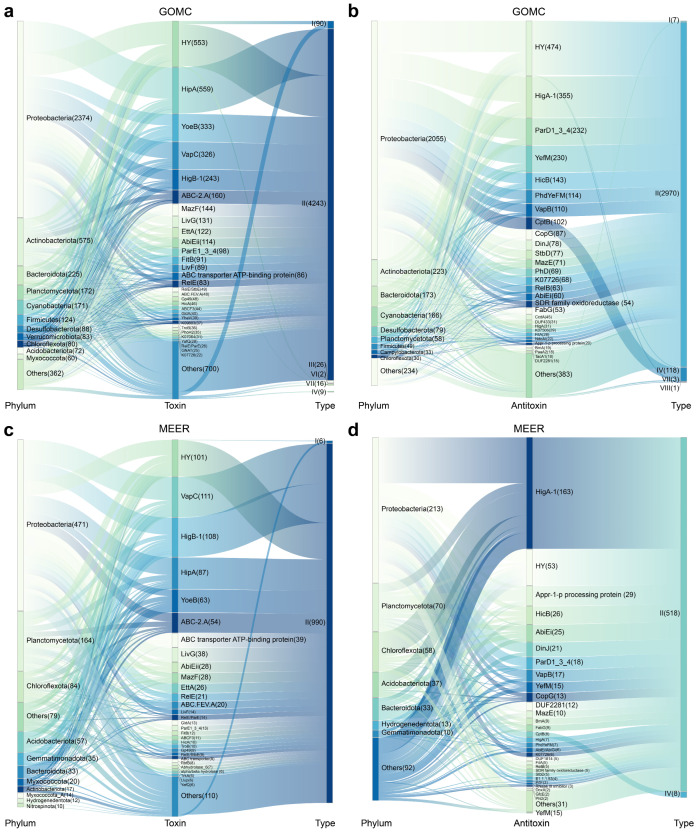
Host-taxonomic associations of marine orphan toxins/antitoxins. Sankey diagrams map host phyla to the abundance of the top 30 orphan toxins (**a**) and orphan antitoxins (**b**) in the GOMC database; corresponding Sankey visualizations of the top 30 orphan toxins (**c**) and orphan antitoxins (**d**) in the MEER database, with connector widths scaled to pairwise association strength; all abundance metrics denote relative proportions.

We next analyzed the distribution of orphan toxins and antitoxins across bacterial taxa. These orphan toxins/antitoxins occur across multiple phyla, with *Proteobacteria*, *Actinobacteriota*, *Bacteroidota*, *Planctomycetota*, *Cyanobacteria*, *Firmicutes*, *Desulfobacteroa*, *Chloroflexota* and *Acidobacteriota* showing particularly broad distributions in the GOMC (Figure 7a,b). Within the GOMC database, *Proteobacteria* dominated the abundance of orphan elements with 2374 toxins and 2055 antitoxins, followed by *Actinobacteriota* (575, 223) and *Bacteroidota* (225, 173) (Figure 7a,b). Striking distributional asymmetries emerged in the other phyla. The *Verrucomicrobiota* MAGs included 83 orphan toxins but only 18 orphan antitoxins. Furthermore, orphan toxins/antitoxins exceeded cognate TA systems in *Planctomycetota* (172, 58), *Chloroflexota* (80, 30) and *Acidobacteriota* (72, 22) (Figure 7a,b). Interestingly, genomic mining of the GOMC database revealed striking variations in orphan TA element inventories: *Escherichia coli* (GOMC-BGIocean__2704) possessed 26 type I, one type II, and one type IV orphan toxin, alongside six type II and two type I antitoxins. Similarly, *Escherichia* sp. 005843885 (GOMC-BGIocean__2705) contained ten type I and three type IV orphan toxins, together with three type I, five type II, two type IV, and one type VIII antitoxin. *Klebsiella pneumoniae* (GOMC-BGIocean__2701) carry eight orphan toxins and two antitoxins, and *Desulfosarcina ovata* (GOMC-BGIocean__16272) carry two orphan toxins and ten antitoxins, respectively. Although *Proteobacteria* and *Planctomycetota* harbored abundant orphan toxins/antitoxins in the MEER MAGs, the phylum-level distributions differed between the GOMC and MEER MAGs (Figure 7c,d). Within the MEER database, the abundances of *Chloroflexota* and *Acidobacteriota* replaced *Actinobacteriota*, and *Cyanobacteria* became the top five phyla in terms of both orphan toxins and antitoxins. These orphan toxins/antitoxins were also identified in *Gemmatimonadota* (35, 10) and *Hydrogendentota* (12, 13) (Figure 7c,d). Within the MEER database, orphan toxins/antitoxins generally presented low-abundance profiles similar to those of canonical TA systems. Representative examples include *Pseudorhizobium pelagicum* (MEER__5422), which contains seven toxins and three antitoxins, and *Allorhizobium rosettiformans* (MEER__1889), which contains six toxins and five antitoxins. Notably, a single MAG (MEER__5696; *Thermoanaerobaculia*) encoded only five type II orphan toxins. Owing to substantial sequence and structural divergence in their counterparts, our homology-based approach could not preclude the existence of novel orphan toxins/antitoxins with unique architectures or functions. Furthermore, the intriguing prevalence of orphan toxins/antitoxins warrants future investigation into their genomic context, specifically to determine if they are co-located with plasmids, prophages, or other mobile genetic elements, which could elucidate their origins and functional maintenance.

These findings confirm the pervasive dominance of type II TA systems across all oceanic zones. In pelagic ecosystems, complete type II TA systems (YoeB/YefM, HigB/HigA, ParE/ParD, VapC/VapB) predominate, whereas the hadal zone exhibits inverted abundance patterns favoring the orphan components of these same systems. This depth-dependent divergence, characterized by elevated orphan:canonical ratios in extreme environments, likely reflects adaptive evolutionary specialization. Specifically, the enrichment of stress-responsive orphans (e.g., HigA/VapC for genomic stabilization) may enable genome streamlining under high-pressure, oligotrophic conditions. The high taxonomic novelty of the MEER database (89.4% new species among 7564) [21] suggests that these novel lineages may encode equally novel TA systems and orphan toxins/antitoxins, which may exhibit low sequence homology with known TA systems. Functionally, TA systems require antitoxins to inhibit their cognate toxins and prevent self-toxicity. It follows that the antitoxins neutralizing the observed orphan toxins likely escaped detection, possibly due to their unidentified sequence or domain characteristics, and they could be adjacent to the toxins or on mobile genetic elements. Orphan antitoxins may act as transcriptional regulators [40,41], or their toxin partners may be similarly unidentified. Overall, the prevalence of these orphan toxins/antitoxins points to a broad reservoir of novel TA systems with distinct architectures and functions, necessitating further characterization in the hadal zone.

## 3. Materials and Methods

### 3.1. Bacterial Strains, Plasmids and Growth Conditions

The bacterial strains, plasmids and all primers used in the study are listed in Tables S3 and S4. *E. coli* strains were cultured at 37 °C with shaking at 220 rpm in Luria–Bertani (LB) medium. Chloramphenicol (30 μg/mL) was used to maintain the pTac plasmids. IPTG (0.2 mM) was added to induce gene expression when needed.

### 3.2. Marine Metagenome Sequence Data Collection

GOMC (https://db.cngb.org/maya/datasets/MDB0000002; accessed on 25 September 2025) contains 24,195 species-level genomes spanning diverse marine environments (77.90° S–89.99° N; surface to 10,000 m depth). MEER (https://www.biosino.org/node/project/detail/OEP004067; accessed on 25 September 2025) provides 7564 metagenome-derived species-level representative genomes of hadal ecosystems. Prokaryotic genomes from both repositories were downloaded to systematically predict the TA system distribution in marine organisms. All the genomes were renamed before analysis because of the integrated datasets in GOMC (NCBI [42], OMD (https://microbiomics.io/ocean/; accessed on 25 September 2025) [23], and OceanDNA [43]) (Tables S7 and S8).

### 3.3. TA Prediction

The genomes were analyzed via TADB 3.0 with TAfinder 2.0 (https://bioinfo-mml.sjtu.edu.cn/TADB3/TAfinder.php; accessed on 25 September 2025) for TA system identification [34]. InterProScan 106.0 [44] and HMMER v3.4 [45] were used to predict the domains of hypothetical toxin/antitoxin proteins. Similar proteins were identified via NCBI BLASTp 2.16.0 [46] for each toxin/antitoxin. Detailly, to identify TA systems, the predicted genes were queried against the TADB 3.0 database using BLASTn 2.16.0. A hit was considered a valid TA system if it met the following criteria: (1) amino acid identity > 70%; (2) e-value < 1 × 10^−5^; (3) alignment coverage (alignment length/subject length) > 50%; (4) gene length between 30 and 500 amino acids; and (5) an intergenic spacer between the TA systems ranging from −20 to 150 bp. Orphan toxins or antitoxins were defined as genes that satisfied criteria (1) to (4) but were not part of any identified TA systems, with the additional requirements that the gene (5) was not located at the contig edge and (6) was absent from the paired-TA results. All annotated toxins and antitoxins were verified that they contain the same Pfam domains as their corresponding identified homologues. To validate the prediction workflow, we applied it to one well-annotated reference genomes, *E. coli* K-12 substr. MG1655 (NCBI accession number: NC_000913), using 37 published TA systems. The workflow achieved recall rate of 97.30% (36/37) and potential false positive rate of 2.70% (1/37) (Table S9). The predicted structures of RelE from *Marinomonas* sp. and VapC from *SIO2C1* sp. were generated via AlphaFold3 [47]. Structure alignment and related images were generated via PyMOL 3.1. All the phenotypes of the MAGs listed in Table S10 were predicted via Traitar v1.1.2 [48].

### 3.4. Bibliometric and Geospatial Analysis

Abstracts of TA system publications (Web of Science, 1983–2025) were retrieved. Annual and cumulative publication trends were visualized via GraphPad Prism 9.5. Sankey diagrams mapping TA systems to physiological functions or host classifications were generated with ChiPlot (https://www.chiplot.online/; accessed on 25 September 2025). The geographical coordinates of the TA-containing genomes sourced from GOMC and OMD were mapped via ChiPlot. All the bar charts and partial databases were processed in GraphPad Prism 9.5.

### 3.5. Plasmid Construction

For the genomic predictions, two novel TA combinations were prioritized for functional validation. The TA operons were synthesized (Tsingke Biotech, Beijing, China) and amplified via PCR with the primers listed in Table S4. The purified PCR products were ligated into EcoRI/BamHI-digested pTac and then transformed into *E. coli* MG1655. The expression of TA genes was under the control of the IPTG-inducible promoter.

### 3.6. Toxicity Assay of the TA System

TA system functionality was evaluated through IPTG-inducible expression in *E. coli* MG1655. Overnight cultures of *E. coli* strains harboring the above plasmids were diluted to OD_600_ ~ 0.01 in fresh medium. Expression was induced with 0.2 mM IPTG. Growth kinetics were monitored in 200 μL cultures via 96-well plates (37 °C, orbital shaking), with the OD_600_ recorded at 1 min intervals. For each experiment, three biological replicates are presented as individual curves, and each represents the means ± SDs of three technical replicates.

## 4. Conclusions

Our integrated analysis reveals an unprecedented diversity of TA systems in marine microorganisms, featuring noncanonical architectures and evolutionary innovations distinct from those of established model organisms. The identification of 295 HY/HY domain pairs, unconventional combinations (e.g., ParE/CopG and RelE/StbD), and widespread orphan toxins/antitoxins underscores the role of extreme marine habitats, particularly the deep-sea, in driving TA system specialization. These divergent configurations, which include toxins coupled with uncharacterized antitoxin domains and hybrid PIN–RNA/DNA-binding domains, likely represent adaptive responses to high pressure, nutrient limitation, and oxidative stress. Despite a marked decline in classical TA system abundance at the community level in hadal zones compared to shallower regions, it remains possible that antitoxins or toxins with unidentified molecular characteristics exist adjacent to the predicted orphan toxins or antitoxins. Indeed, this is supported by the observation that certain species exhibited unexpectedly high numbers of TA systems. These genomic features likely evolved in response to the strong selective pressures of the deep-sea, which drive the enrichment of genes involved in biofilm formation, antibiotic resistance, and antioxidation to counter reactive oxygen species (ROS) [21]. In this context, we posit that novel TA systems may play an integral role in regulating these critical adaptive mechanisms. We further propose that the heterogeneity in TA system abundance at the species level can be accounted for by dynamic, microenvironment-specific stresses, such as horizontal gene transfer, viral infection, and nutrient limitation.

Despite these advances, systematic exploration of TA systems continues to face significant challenges. Relying on sequence homology, our analysis is inherently constrained in predicting novel toxins/antitoxins that exhibit significant sequence divergence from known references. This approach favors well-characterized systems, explaining the high enrichment of type II TA systems, which have been most extensively documented. Due to the intrinsic diversity of TA systems, especially those involving RNA antitoxins and/or toxins (types I, III, and VIII), sequence homology-based prediction hampered accurate evaluation of TA abundance and distribution. This approach inherently underestimates noncanonical systems, as RNA-based elements often lack conserved sequence motifs and depend on secondary structures or interaction contexts that are not captured by conventional alignment tools. Furthermore, metagenomic identification is constrained by fragmented contigs and high sequence divergence. Functional validation of the predicted TA systems or orphan toxins/antitoxins is further hindered by the uncultivability of many deep-sea microbial phyla and a general lack of genetic tools for these environmentally specialized lineages. Crucially, while these limitations may affect absolute quantifications, the uniform application of our analytical pipeline across all samples ensures that the comparative trends central to our conclusions remain convincing.

Our study extends beyond unconventional TA systems (Table 2) to encompass a widespread distribution of orphan toxins/antitoxins across marine habitats. Novel TA system types such as type VII and type VIII were identified in specialized lineages [17,49], suggesting that MEER database may offer a substantial reservoir of uncharacterized TA systems in extreme marine environments. Furthermore, the proposed biological roles of orphan antitoxins, such as in transcriptional regulation or toxin neutralization, require direct experimental confirmation. Consequently, resolving their genomic context, particularly any association with mobile elements, is critical for reconstructing their origins and evolutionary maintenance.

To overcome these limitations, future efforts should adopt integrative, structure-aware strategies. Prediction methods combining structural similarity with gene neighborhood analysis can uncover evolutionarily conserved TA systems beyond sequence homology. Tools analyzing genomic co-occurrence networks [50] may identify novel TA types with divergent sequences but conserved functions. Machine learning models integrating sequence, structure, and genomic context could further improve TA detection and functional inference in uncultured marine taxa. These approaches would help prioritize candidates for validation and illuminate the adaptive mechanisms of marine TA systems, accelerating their biotechnological application.

## Figures and Tables

**Table 1:** Catalog of TA systems and their microbial hosts in the GOMC and MEER databases. Sampling depths are provided for MAGs sourced from the MEER project. Archaeal MAGs are in blue. Species with no existing entries in TADB 3.0 are indicated in bold. The prefix “GOMC-BGIocean” of MAG is only abbreviated as “GOMC” in this table.

MAG (Depth)	Species (Phylum)	TA Number	TA Annotation (Number)
GOMC_5042	*Marinobacter* sp. Arc7-DN-1 (*Proteobacteria*)	18	Diaminopimelate decarboxylase/HY, HigB/HigA, HipA/HipB, K09803/CopG, HigB-1/HigA-1 (2), ParE1_3_4/ParD1_3_4 (2), RelE/HY, YoeB/YefM (4), ParE1_3_4/PhD (2), StbE/HY, StbE/StbD, VapC/VapB
GOMC_2532	*Vibrio cholerae* (*Proteobacteria*)	17	Doc/HY, DUF4160/DUF2442, GCN5/TacA1, HigB-1/DUF6946, HigB-1/HigA-1, HipA/HY, HY/DinJ, HY/DUF1778 (2), ParE/PhdYeFM, ParE1_3_4/ParD1_3_4, ParE1_3_4/PhD, K07726/RsaL, StbE/StbD (2), YafQ/DinJ, YafQ/HY
GOMC_2698	*Pantoea agglomerans* (*Proteobacteria*)	17	Diaminopimelate decarboxylase/HY, Hha/TomB, HicA/HicB, HipA/HY (2), HipA/HipB, HY/HY, HY/TacA1 (2), HigB-1/HigA-1, K07726/HY, RelE/HY, RelE/RelB, StbE/StbD, VapC/VapB, YhaV/PrlF, YkfI/YafW
GOMC_16272	*Desulfosarcina ovata* (*Desulfobacterota*)	14	Gp49/RelB (2), HicA/HicB, HipA/HY, HY/HY, HY/Mnt, MazF/MazE, ParE1_3_4/PhdYeFM, PilT/PhdYeFM, VapC/VapB (2), YafQ/DinJ, YoeB/YefM (2)
**GOMC_2702**	***Microcystis panniformis_A*** **(*Cyanobacteria*)**	12	CbtA/CbeA, CptA/CptB, Hha/TomB, HigB/HigA, HY/TacA1, HY/CopG (2), K07726/HY, K09803/YhbS (2), MazF/HY (2), RelE/HY, VapC/VapB
**GOMC_13019**	***Mycobacterium poriferae*** **(*Actinobacteriota*)**	5	HigB-1/HigA-1, HY/HY, K07064/HY, MazF/MazE, VapC/HY
GOMC_22898	*Thermococcus* sp. 900198835 (*Methanobacteriota*)	4	K07064/MazE (3), K07064/CcdA (1)
GOMC_22906	*Thermococcus indicus* (*Methanobacteriota*)	4	K07064/MazE (2), VapC/CcdA (2)
GOMC_22189	*Archaeoglobus fulgidus* (*Halobacteriota*)	2	VapC/MazE, HY/MazE
MEER__7415 (6384.4 m)	*Acinetobacter*; *s__* (*Proteobacteria*)	16	BrnT/BrnA, HigB/HigA, HY/HY, K07726/HY (2), K09803/HY (4), VapC/VapB (2), ParE1_3_4/PaaA2, RelE/HY, RelE/RelB (2), YoeB/YefM
**MEER__7417** **(6384.4 m)**	***Bythopirellula*** **; *s__*** **(*Planctomycetota*)**	3	HicA/HicB, RelE/HicB, RelE/RelB
MEER__7435 (6384.4 m)	*Citrobacter freundii* (*Proteobacteria*)	11	CbtA/CbeA (3), CcdB/CcdA (2), CptA/CptB, Doc/PhD, Hha/TomB, HY/HY (2), RelE/HY
**MEER__515** **(7725 m)**	***Halomonas aquamarina*** **(*Proteobacteria*)**	4	HigB-1/HigA-1, HY/HY, HY/ParD1_3_4, K09803/HY
**MEER_518** **(7725 m)**	***Alishewanella agri*** **(*Proteobacteria*)**	4	HY/HY (2), K07726/RsaL, StbE/StbD
**MEER__5075** **(8394.2 m)**	***Sphingobium limneticum*** **(*Proteobacteria*)**	6	K09803/BrnA, ParE1_3_4/DinJ, ParE1_3_4/HY, ParE1_3_4/ParD1_3_4, RelE/HY, VapC/VapB
**MEER__3888** **(8868.9 m)**	***Pseudomonas_E oleovorans*** **(*Proteobacteria*)**	6	ArgA/AraC, FitB/Phd, ParE1_3_4/CopG (2), ParE1_3_4/PaaA2, ParE1_3_4/PhdYeFM
**MEER__5422** **(9454 m)**	***Pseudorhizobium pelagicum*** **(*Proteobacteria*)**	6	VapC/VapB, FitB/DinJ, HipA/HipB, ParE1_3_4/FitA, ParE1_3_4/ParD1_3_4, FitB/FitA
**MEER__5469** **(9556 m)**	***Acinetobacter idrijaensis*** **(*Proteobacteria*)**	8	CptA/CptB, Gp49/HY (2), K07726/HY, K09803/HY (2), RelE/RelB, VapC/VapB
**MEER__2668** **(10,026.2 m)**	***f__UBA1845*** **(*Planctomycetota*)**	1	DUF4433/Macro
**MEER__4515** **(10,888.46 m)**	***o__SZUA-224*** **(*Nitrospinota*)**	1	VapC/VapB

**Table 2:** Canonical and novel combination of TA systems identified in the GOMC and MEER databases. The experimentally validated TA systems are indicated in bold. The primary domains of hypothetical toxins/antitoxins are listed.

Canonical	Novel	Description of HY
	HY/HY (295)	T: RES, PIN, HD_3 A: dnstrm_HI1420, IclR, HTH, Xre_MbcA_ParS
AbiEii/AbiEi (100)	AbiEii/HY (9)	
	AbiEii/DUF6088 (13)	
BrnT/BrnA (5)	K09803/BrnA (28)	
Doc/PhD (10)	Doc/HY (16)	Acetyltransf_10, HTH
	Doc/DUF6290 (3)	
FitB/FitA (62)	FitB/Phd (22)	
	FitB/PhdYeFM (12)	
HigB-1/HigA-1 (302)	HY/HigA-1 (15)	HigB-like, HTH_18
HigB/HigA (33)	HigB/HY (4)	HTH_3, SpoIVA_ATPase
	HigB-1/HY (4)	HTH_3, rve_3, dnstrm_HI1420
	K07726/HigA-2 (10)	
HipA/HipB (161)	HipA/HY (81)	HTH_3, HTH_31, DUF1707
HicA/HicB (119)	HicA/UPF0150 (3)	
	HY/HicB (2)	
MazF/MazE (87)	HY/MazE (41)	PIN
	MazF/HY (11)	HTH_RNase_II, HTH_3
	Nucleic acid-binding protein/MazE (11)	
	VapC/MazE (8)	
	MazF/PrlF (5)	
MqsR/MqsA (19)	K07726/MqsA (41)	
ParE1_3_4/ParD1_3_4 (250)	ParE1_3_4/HY (27)	RHH_1, RHH_9
	ParE1_3_4/PhdYefM (136)	
	ParE1_3_4/PhD (118)	
	ParE1_3_4/DinJ (117)	
	ParE1_3_4/CopG (33)	
	ParE1_3_4/RelB (18)	
	ParE1_3_4/PaaA2 (15)	
	HY/ParD-like (6)	
RelE/RelB (66)	**RelE/HY (112)**	HTH_37, HTH_3, **dnstrm_HI1420**
	Gp49/RelB (14)	
	RelE/StbD (10)	
	RelE/DinJ (7)	
	RelE/CopG (6)	
StbE/StbD (161)	StbE/HY (20)	HTH_3, phage_T7_Gp5.9
	StbE/YefM (17)	
	StbE/PhdYefM (13)	
	StbE/CopG (5)	
VapC/VapB (245)	HY/VapB (36)	PIN
	VapC/CopG (8)	
	VapC/MazE (8)	
	VapC/PhdYeFM (7)	
	**VapC/DUF2281 (5)**	
YafQ/DinJ (57)	K09803/DinJ (5)	
YoeB/YefM (509)	YoeB/HigA-1 (3)	

## Data Availability

The data used in this study are available in the published article and its Supplementary Materials.

## Supplementary Materials

The following supporting information can be downloaded at https://www.mdpi.com/article/10.3390/md23110436/s1. Table S1: Summary of the predicted TA systems in the GOMC and MEER databases (*n* = 5086); Table S2: Sequences of the validated TA systems; Table S3: Bacterial strains and plasmids used in this study; Table S4: Oligonucleotides used for gene cloning and DNA sequencing; Table S5: Summary of the predicted orphan toxins in the GOMC and MEER databases (*n* = 5382); Table S6: Summary of the predicted orphan antitoxins in the GOMC and MEER databases (*n* = 3625); Table S7: Corresponding names of the analyzed MAGs based on the GOMC database; Table S8: Corresponding names of the analyzed MAGs based on the MEER database; Table S9: List of predicted TA pairs in *Escherichia coli* K-12 MG1655 (NCBI accession number: NC_000913); Table S10: Predicted phenotypes of the TA-containing MAGs via Traitar v1.1.2.

## Abbreviations

The following abbreviations are used in this manuscript:

TA	Toxin–antitoxin
MGEs	Mobile genetic elements
MAGs	Metagenome-assembled genomes
GOMC	Global Ocean Microbiome Catalogue
MEER	Mariana Trench Environment and Ecology Research
HHP	Hydrostatic pressure
LT	Low temperature
OMD	Ocean Microbiome Database
LB	Luria–Bertani
ROS	Reactive Oxygen Species
