# Supplementary material for: The Prevalence and Diversity of Marine Toxin–Antitoxin Systems
Source: Mar Drugs. 2025 Nov 13;23(11):436. doi: 10.3390/md23110436 (PMC12654330; doi:10.3390/md23110436)
Supplement: Supplementary file 1 [file marinedrugs-23-00436-s001.zip › 20250930-Supplementary Material for Marine Toxin-Antitoxin Systems.pdf]

Supplementary Information for

Marine Toxin-Antitoxin Systems: From Genomic Landscape to Adaptive Significance

Cong Liu [1,2], Yunxue Guo [1,2*], Jiayu Gu [1,2], Zhen Wei [1,2], Pengxiang Chen [1,2], and Xiaoxue Wang [1,2*]

*Corresponding authors. Email: yunxueguo@scsio.ac.cn (Y.G.); xxwang@scsio.ac.cn (X.W.)

The PDF file includes:

Supplementary Tables

Table S1 (available online): Summary of the predicted TA systems in the GOMC and MEER datasets (n = 5,086).

Table S2 (available online): Sequences of the validated TA systems.

Table S3: Bacterial strains and plasmids used in this study.

Table S4: Oligonucleotides used for gene cloning and DNA sequencing.

Table S5 (available online): Summary of the predicted orphan toxins in the GOMC and MEER datasets (n = 5,382).

Table S6 (available online): Summary of the predicted orphan antitoxins in the GOMC and MEER datasets (n = 3,625).

Table S7 (available online): Corresponding names of the analyzed MAGs based on the GOMC dataset.

Table S8 (available online): Corresponding names of the analyzed MAGs based on the MEER dataset.

Table S9 (available online): Predicted phenotypes of the TA-containing MAGs via Traitar.

Supplementary Tables

Supplementary Table S3. Bacterial strains and plasmids used in this study. R indicates resistance, Cm indicates chloramphenicol.

	Description	Source
**Strains**		
MG1655	F-lambda-ilvG-rfb-50 rph-1	lab stored
Top10	F- mcrA Δ(mrr-hsdRMS-mcrBC) φ80 lacZΔM15Δ lacX74 recA1 araΔ139Δ(ara-leu)7697 galU galK rpsL (Str^R)endA1 nupG	Sangon Biotech (Shanghai) Co., Ltd.
**Plasmids**		
pTac	Cm^R, P_{T7} expression vector	lab stored
pTac-*relE*	Cm^R, *relE* in pTac EcoRI/BamHI	this study
pTac-*dnstrm_HI1420*	Cm^R, *dnstrm_HI1420*in pTac EcoRI/BamHI	this study
pTac-*relE-dnstrm_HI1420*	Cm^R, *relE-dnstrm_HI1420* in pTac EcoRI/BamHI	this study
pTac-*vapC*	Cm^R, *vapC* in pTac EcoRI/BamHI	this study
pTac-*DUF2281*	Cm^R, *DUF2281* in pTac EcoRI/BamHI	this study
pTac-*vapC-DUF2281*	Cm^R, *vapC-DUF2281*in pTac EcoRI/BamHI	this study

Supplementary Table S4. Oligonucleotides used for gene cloning and DNA sequencing. F indicates forward primer and R indicates reverse primer.

Primer name	Sequence (5'-3')	Purpose
**Primers for cloning pTac based clones**		
pTac-*relE*-F	CAATTTCACACAGGAGAATTCATGTACGATATTAAACTGACGGA	Construction of pTac-*relE*, pTac-*dnstrm_HI1420* and pTac-*relE-dnstrm_HI1420*
pTac-*relE*-R	AAAACAGCCAAGCTTGGATCCTCATGTCGTCTCATCCAGTTTAATCG	
pTac-*dnstrm_HI1420*-F	CAATTTCACACAGGAGAATTCATGACTAGCATTAAAGAACTGACCAACT	
pTac-*dnstrm_HI1420*-R	AAAACAGCCAAGCTTGGATCCTTATTTCTGGCTATGAATCACCGCT	
pTac-*vapC*-F	CAATTTCACACAGGAGAATTCATGAAATACCTGCTGGACACCC	Construction of pTac-*vapC* and pTac-*DUF2281* and pTac-*vapC-DUF2281*
pTac-*vapC*-R	AAAACAGCCAAGCTTGGATCCTTAGTAATCCCACAGGCGCT	
pTac-*DUF2281*-F	CAATTTCACACAGGAGAATTCATGAACACCGAACTGGCG	
pTac-*DUF2281*-R	AAAACAGCCAAGCTTGGATCCTTACATATATTCCGCCAGATCTTCCAG	
**Plasmid verification primers**		
pTac-F	TTGAGAAGCACACGGTCACACTG	Verification of constructions used for gene expression
pTac-R	TGCGTTCACCGACAAACAACAGATA	
